# Seasonal genetic partitioning in the neotropical malaria vector, *Anopheles darlingi*

**DOI:** 10.1186/1475-2875-13-203

**Published:** 2014-05-29

**Authors:** Aline F Angêlla, Patrícia Salgueiro, Luiz HS Gil, José L Vicente, João Pinto, Paulo EM Ribolla

**Affiliations:** 1 Departamento de Parasitologia, Instituto de Biociências, Universidade Estadual Paulista “Júlio de Mesquita Neto”, Botucatu, SP, Brasil; 2 Centro de Malária e outras Doenças Tropicais/UEI Parasitologia Médica, Instituto de Higiene e Medicina Tropical, Universidade Nova de Lisboa, Lisboa, Portugal; 3 IPEPATRO- Instituto de Pesquisas em Patologias Tropicais, Porto Velho, RO, Brasil

**Keywords:** *Anopheles darlingi*, Amazonia, Seasonal genetic structure, Microsatellites, Malaria

## Abstract

**Background:**

*Anopheles darlingi* is the main malaria mosquito vector in the Amazonia region. In spite of being considered a riverine, forest-dwelling species, this mosquito is becoming more abundant in peri-urban areas, increasing malaria risk. This has been associated with human-driven environmental changes such as deforestation.

**Methods:**

Microsatellites were used to characterize *A. darlingi* from seven localities along the Madeira River, Rondônia (Brazil), collected in the early and late periods of the rainy season.

**Results:**

Two genetically distinct subpopulations were detected: one (subpopulation A) was associated with the late rainfall period and seems to be ecologically closer to the typical forest *A. darlingi*; the other (subpopulation B) was associated with the early rainfall period and is probably more adapted to drier conditions by exploiting permanent anthropogenic breeding sites. Results suggest also a pattern of asymmetric introgression, with more subpopulation A alleles introgressed into subpopulation B. Both subpopulations (and admixed mosquitoes) presented similar malaria infection rates, highlighting the potential for perennial malaria transmission in the region.

**Conclusions:**

The co-occurrence of two genetically distinct subpopulations of *A. darlingi* adapted to different periods of rainfall may promote a more perennial transmission of malaria throughout the year. These findings, in a context of strong environmental impact due to deforestation and dam construction, have serious implications for malaria epidemiology and control in the Amazonian region.

## Background

*Anopheles darlingi* is a major malaria mosquito vector in the Americas. It is of particular importance in the Amazon basin where most malaria cases occur in South America [1,2]. This species has a widespread geographic distribution (from Mexico to Argentina) is highly anthropophilic and is susceptible to human *Plasmodium* malaria parasites. *Anopheles darlingi* is typically described as a lowland, riverine, forest-dwelling species [3]. However, mosquito populations of this species display great behavioural plasticity and morphological, biological and genetic diversity [1,3-6]. Moreover, this vector has been found at high densities in areas affected by deforestation and anthropogenic environmental changes [7].

Due to its intrinsic diversity the question of *A. darlingi* being a species complex has been raised by many authors. Charlwood [8] found chromosomal, morphological and behavioural differences between *A. darlingi* from central Amazon and further north (Venezuela and Guyana). However, several isozyme and DNA-based studies reported levels of genetic differentiation consistent with a monotypic species throughout the species' range [9-11]. Although mtDNA history revealed some genetic divergence between South and Central American populations [12-14], complete mtDNA genomes of *A. darlingi* did not provide support for speciation within the taxon [6]. Nuclear microsatellite markers revealed restricted contemporary gene flow among *A. darlingi* populations over broad geographical ranges (continental scale) but mainly due to isolation by distance [15-17].

Precipitation is considered to be the main ecological variable influencing *A. darlingi* distribution and abundance [3]. The annual density of *A. darlingi* populations is seasonal and dependent upon the availability of larval habitats [1]. In riverine areas, seasonality varies between less populated rural sites, with high mosquito densities after the peak of rainfall [18], and urban and suburban sites, where two density peaks may occur in the transition from the dry to the rainy season and *vice versa*[19,20]. In drier inland areas (further from the influence of rivers) with human disturbance, a peak of mosquito density may occur in the dry season, associated with the presence of artificial permanent breeding sites, as dams and artificial lakes [18,21].

Variable density peaks may be the result of seasonal fluctuations of the same population or the consequence of the presence of two differentially adapted subpopulations of the same species. In both cases, there is a growing concern that the increased anthropogenic influence in riverine, forested habitats of *A. darlingi* may result in changes in the seasonality patterns of this mosquito with implications for malaria transmission. Such is the case of the region of Porto Velho, in Rondônia state, Brazil. This city lies along the Madeira River, an important tributary of the Amazona River. A major environmental disturbance is expected in this region due to the construction of two hydroelectric power dams along the river, with a planned total flooded area of 380 sq km, which will likely impact local mosquito populations.

In the present study, microsatellite loci were used to analyse genetic variation of Amazonian *A. darlingi* from the region of Porto Velho (RO). Results suggest a season-dependent genetic partition, with two distinct mosquito subpopulations that display similar *Plasmodium* infection rates.

## Methods

### Mosquito collections

**Figure 1:**
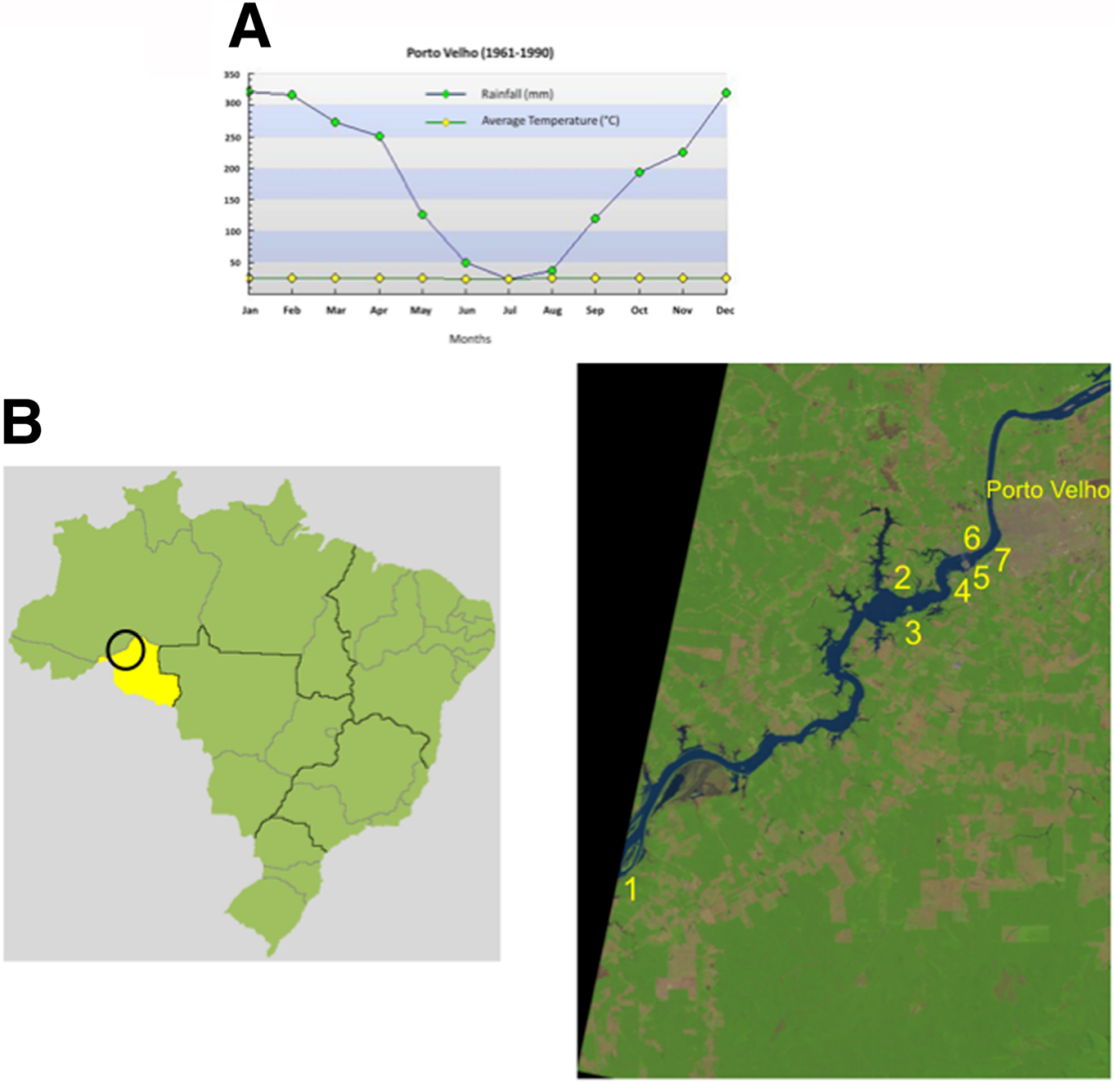
**Porto Velho mean annual rainfall and average temperature values between 1961 and 1990 (A) and Map of Brazil, showing de state of Rondonia and the collection sites along the Madeira River (B).** Rainfall and temperature data was obtained from Instituto Nacional de Meteorologia (INMET). The Landsat image was obtained from http://glovis.usgs.gov/ (U. S. Geological Survey). 1: Jaci Parana; 2: Amazonas; 3: Teotonio; 4: Bate Estaca; 5: Santo Antonio; 6: Engenho Velho; 7: Vila Candelaria.

Porto Velho (*ca.* 436,000 inhabitants) is the capital city of the state of Rondônia (Brazil), which lies along the shores of Madeira River in the western part of the Amazonian forest, close to the border with Bolivia. Most of the human population lives on the right bank of the Madeira River, while the left bank is sparsely populated with small farms and settlements created by the National Institute of Colonization and Agrarian Reform. The state is one of the most affected by deforestation in Brazil [22]. The region has a tropical monsoon climate (Koppen classification, Am) with a dry season from May to September and a rainy season from October to April [23]. *Anopheles darlingi* abundance is seasonal and follows the rainfall regime. During the rainy season, human-biting mosquito densities reach a peak in March-April, with over 30 bites per human per hour, whereas in the dry season biting rates fall to nearly zero [19]. In urban and suburban areas of the region, an additional density peak is recorded between the end of the dry season and the beginning of the next rainy season (October-November) and malaria transmission continues throughout the year [19]. Mean annual rainfall and average temperature values between 1961 and 1990 were obtained for the region from Instituto Nacional de Meteorologia (INMET) (Figure 1A).

Mosquito sampling took place in seven localities along the Madeira River near Porto Velho (Figure 1B; Table 1): Vila Candelária, a riverside district of the city, less than 2 km from the city centre; the suburban riverine localities of Bate Estaca, Engenho Velho, Santo Antônio, Teotônio, and Amazonas, located 5 to 20 km southwest of the city centre; and, Jaci Paraná, a more inland locality located in a deforested area, *ca.* 75 km southwest of Porto Velho.

**Table 1:** **Characterization of the sampling sites of *A. darlingi *** **in Madeira River, Rondônia state, Brazil (2007)**

**Site name**	**Human population in 2007**	**Longitude (W)**	**Latitude (S)**	**River side**	**Collection months early rain**	**N early rain**	**Collection months late rain**	**N late rain**
Vila Candelária	335	63°55'00*.*5"	08°47'17,8"	Right	Oct., Dec.	35	Feb., Apr.	43
Bate Estaca	117	63°55'48*.*9"	08°47'55,5"	Right	Oct., Dec.	31	Feb., Apr.	48
Engenho Velho	141	63°56'40*.*3"	08°47'36,4"	Left	Oct., Dec.	39	Feb., Apr.	43
Santo Antônio	224	63°56'34*.*3"	08°48'34,6"	Right	Oct., Dec.	29	Feb., Apr.	48
Teotônio	251	64°03'42*.*0''	08°51'39,5''	Right	n.d.	-	Feb., Apr.	31
Amazonas	n.a.	64°03'50*.*4''	08°51'10,2''	Left	n.d.	-	Feb.	21
Jaci Paraná	2826	64°24'16*.*0"	09°15'30,0"	Inland	Oct.	24	Feb., Apr.	48

Data for the estimates of human population, Geographic coordinates; Total N: total number of individuals analysed with microsatellites; N 1st/2nd sem.: number of individuals analysed per semester; n.d.: not done.

Collections took place between 2007 and 2008 during two different periods of the rainy season: February and March (hereafter termed late rainfall period) corresponding to the end of the rainy season and when *A. darlingi* abundance reaches its peak in the region [19]; October and December (hereafter termed early rainfall period) corresponding to the end of the dry season and the beginning of the rainy season, when mosquito abundance begins to increase. Mosquitoes were captured by outdoor landing catches with hand-held aspirators. Six 6-hour (18.00 to 00.00) collections were performed at each site.

Collected mosquitoes were morphologically identified to species using the identification keys of the subgenus of *Nyssorhynchus*[24]. *Anopheles darlingi* specimens were preserved individually in tubes with 70% isopropyl alcohol.

### Microsatellites genotyping

DNA was extracted from each mosquito (whole body) using 5% Chelex solution (BioRad), according to the manufacturer's recommendations and eluted in 200 μl of double-distilled water.

Ten microsatellite loci were genotyped (see Additional file 1). Eight of the primer pairs were originally described by Conn *et al.*[25]. Due to amplification problems, the primers of locus ADC107 were redesigned. Furthermore, primers for two additional loci (ADMP2 and ADMP9) were obtained from the GenBank sequences reported by Calvo *et al.*[26], both from 3’UTR of unknown transcripts. Each locus was amplified by PCR using fluorescently labelled (FAM, NED, or HEX; Applied Biosystems) reverse primers. PCR reactions were conducted in a 20-μl PCR mix containing 1× PCR GoTaq® Flexi Buffer (Promega), 1.5 mM MgCl_2_, 200 μM dNTPs, 0.5 μM of each primer, 0.5 U of Taq polymerase and 1 μl of DNA template. Cycling conditions included an initial denaturation at 94°C for 5 min, followed by 30 cycles at 94°C for 30 sec, annealing (52-57°C, Additional file 2) for 30 sec, elongation at 72°C for 35 sec; and a final extension step of 5–10 min at 72°C. Amplified fragments were separated by capillary electrophoresis in an automatic sequencer (ABI3730, Applied Biosystems) and sizes scored using the software GeneMarker (SoftGenetics) and GeneMapper (Applied Biosystems).

### *Plasmodium* detection

The detection of *Plasmodium sp.* DNA in individual *A. darlingi* mosquitoes was performed by real-time PCR using a universal primer (P1 forward primer [5’ -ACGATCAGATACCGTCGTAATCTT-3’]) and the species-specific nucleotide sequences of the 18S rRNA genes of *Plasmodium falciparum* (5’-CAATCTAAAAGTCACCTCGAAAGATG-3’) and *Plasmodium vivax* (5’-CAATCTAAGAATAAACTCCGAAGAGAAA-3’) [27]. Each 15-μl reaction mix contained 2 μl of sample DNA, 7.5 μl of 1× DNA SYBR Green reagent, and 0.5 μM concentrations of each primer. The PCR conditions consisted of 50°C for 2 min, followed by 95°C for 10 min and 40 cycles each with 15 sec at 95°C and 1 min at 60°C. Positive samples, when difference from blank Ct was greater than 15 cycles, were re-analysed for confirmation. All positive samples were confirmed.

### Microsatellites data analysis

Estimates of expected heterozygosity (*He*) allele richness (*Rs*) and private allele richness (*Ps*) were calculated in FSTAT v 2.9.3.2 [28] and HP-RARE [29]. Hardy-Weinberg (HW) equilibrium tests were performed with the software ARLEQUIN 3.1.1 [30]. Linkage disequilibrium (LD) between pairs of loci was tested using the log likelihood ratio statistic available in GENEPOP [31,32]. The software MICRO-CHECKER [33] was used to detect the presence of null alleles.

The genetic structure of the *A. darlingi* populations was assessed by computing locus-specific and pairwise *F*_*ST*_ estimates [34] and by analysis of molecular variance (AMOVA) [35], both available in Arlequin. Permutation-based statistical tests were performed with 5,000 permutations. Bayesian clustering analysis with STRUCTURE 2.3.4 [36] was also performed, without prior information on sampling groups, using the admixture model and assuming correlated allele frequencies among populations (λ was set at 1, default value). Twenty independent runs with 10^5^ burn-in steps and 10^5^ iterations were done for each value of *K* (*K* varied from 1 to 13). The number of genetic clusters (*K*) was inferred using the Δ*K* statistic [37] with the software STRUCTURE HARVESTER [38]. The information from the outputs of each *K* (20 runs) was compiled by the Greedy method implemented in CLUMPP software [39]. Individual assignment to clusters was performed using a probability threshold (*Tq*) of 0.80 following the recommendations of Vaha and Primmer [40].

The Bayesian method implemented in NewHybrids [41] was used to estimate the posterior probability of individuals falling into six genotypic classes: pure-bred, F1, F2 and backcrosses. The approach of uniform priors was used. Results were based on the average of five independent runs of 10^5^ iterations. Individual assignment to classes was based on a posterior probability threshold, *Tq >* 0.5.

In order to detect recent population perturbations, Wilcoxon signed-ranks tests of heterozygosity were performed with BOTTLENECK 1.2.02 [42]. Calculations were done under the stepwise mutation model (SMM) and a two-phase model (TPM) with 5% of indels larger than one repeat [43]. Analyses were based on 1,000 replications. Wilcoxon signed-ranks tests to compare means and Chi-square or exact Fisher’s tests on contingency tables were performed with IBM SPSS Statistics v. 22 for Windows (IBM Corp). Whenever multiple tests were performed, the nominal significance level (α = 0.05) was adjusted by the sequential Bonferroni procedure [44].

## Results

A total of 440 *A. darlingi* were analysed in this study. Of these, 158 were collected in the early rainfall period whereas 282 were collected in the late rainfall period (Table 1).

### Genetic diversity

All ten microsatellite loci were polymorphic and a total of 212 alleles were detected across all loci and samples. The number of alleles per locus ranged from five (ADSP2) to 44 (ADC01). Exact tests of LD showed one significant association for the pair ADC29-ADC110 in the sample of Amazonas, early rainfall period (*P* <0.001; adjusted significance level for 45 pairwise tests per population). Significant departures from H-W equilibrium were detected at loci ADC107n (all samples) and ADC29 (in 11 samples) suggesting the presence of null alleles (Table 2). These loci were thus excluded from subsequent analyses.

**Table 2:** **Microsatellite variation for *A. darlingi *** **in Porto Velho (RO)**

**Rainy season**	**Site name N**	**He Rs**	***Loci***									
			**ADC28**	**ADC29**	**ADC01**	**ADC110**	**ADC138**	**ADC02**	**ADC137**	**P2**	**107n**	**P9**
Late	Amazonas 1	He	0*.*801	**0 *. *** 842*	0*.*958	0*.*820	0*.*751	0*.*815	0*.*832	0*.*408	**0 *. *** 867*	**0 *. *** 864*
	N = 21	Rs	7	11	21	10	7	10	7	4	9	9
Late	Bate Estaca 1	He	0*.*771*	**0 *. *** 634*	0*.*934	0*.*849*	**0 *. *** 872*	0*.*795	**0 *. *** 865*	0*.*274*	**0 *. *** 871*	**0 *. *** 785*
	N = 48	Rs	7	8	17	9	11	10	11	3	11	7
Early	Bate Estaca 2	He	**0 *. *** 843*	**0 *. *** 851*	0*.*955	**0 *. *** 812*	**0 *. *** 897*	0*.*745	0*.*889	0*.*037	**0 *. *** 837*	0*.*650
	N = 31	Rs	8	14	21	8	12	9	10	2	9	7
Late	Vila Candelária 1	He	**0 *. *** 696*	**0 *. *** 762*	**0 *. *** **948**	0*.*841	0*.*876*	0*.*792	0*.*857	0*.*263	**0 *. *** 872*	**0 *. *** 747*
	N = 43	Rs	7	11	19	8	10	10	10	2	9	8
Early	Vila Candelária 2	He	**0 *. *** 832*	**0 *. *** 795*	0*.*941	**0 *. *** 786*	**0 *. *** 894*	**0 *. *** 809*	0*.*869*	**0 *. *** **033**	**0 *. *** 865*	**0 *. *** 558*
	N = 35	Rs	9	11	19	6	11	12	9	2	11	7
Late	Engenho Velho 1	He	0*.*776	**0 *. *** 755*	0*.*935	0*.*844	**0 *. *** 873*	0*.*797	0*.*891	0*.*155	**0 *. *** 848*	0*.*820
	N = 43	Rs	8	11	17	9	11	9	10	3	10	9
Early	Engenho Velho 2	He	0*.*827	**0 *. *** 774*	0*.*944*	0*.*826*	**0 *. *** 884*	0*.*835	0*.*881	0*.*075*	**0 *. *** 829*	0*.*659*
	N = 35	Rs	9	11	17	7	12	10	10	2	9	8
Late	Jaci Paraná 1	He	0*.*807*	**0 *. *** 752*	0*.*946	0*.*831	**0 *. *** 889*	0*.*816	0*.*877*	**0 *. *** **295**	**0 *. *** 856*	**0 *. *** 866*
	N = 48	Rs	8	11	18	9	11	11	9	4	10	11

N: sample size; He: Expected heterozygosity; Rs: Allelic Richness per locus and population based on min. sample size of 20 diploid individuals; In bold: significant P-value after Bonferroni correction. * Presence of null alleles as estimated by Micro-checker.

Estimates of expected heterozygosity (*He*) and allelic richness (*Rs*) per locality and sampling period are presented in Table 2. The mean *Rs* over loci ranged from 9 to 10. The mean *He* over loci ranged from 0.142 in Jaci Paraná to 0.838 in Santo Antônio (both early rainfall periods). The largest difference in *He* between sampling periods was found in the most inland sample of Jaci Paraná, with an estimate of 0.142 in the early rainfall and 0.794 in the late rainfall.

### Genetic structure

**Figure 2:**
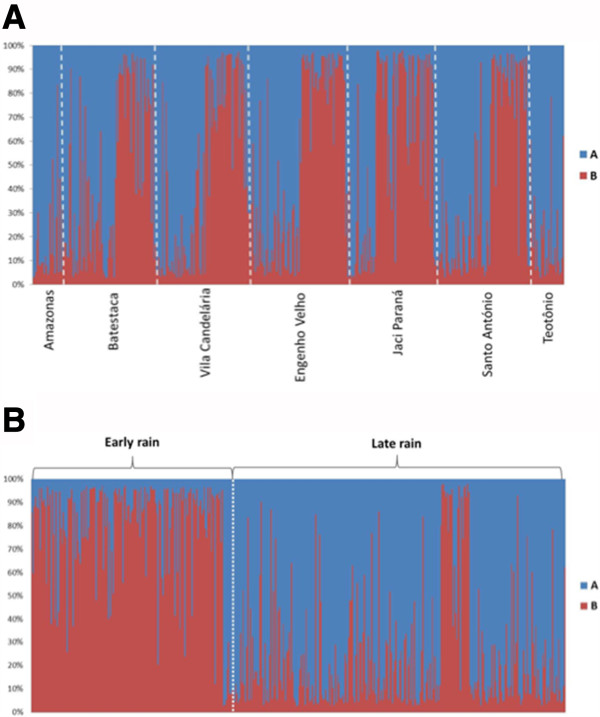
**Bayesian clustering analysis by STRUCTURE [**[36]**] of *A. darlingi.*** The multilocus genotype of each individual is represented by a column with two colours showing the genetic proportion assigned to each cluster (*K* = 2). Blue: Lineage **A**; Red: Lineage **B**. Individuals were ordered by locality and sampling period (ER: early rain; LR: late rain).

Bayesian clustering analysis implemented in STRUCTURE revealed two genetically distinct subpopulations (best *K* = 2), hereafter denoted as subpopulations A and B (Figure 2). A total of 133 individuals showed admixed ancestry between the two subpopulations (i e, 0.2 < *qi* < 0.8). When these individuals were excluded and subpopulations A and B were analysed separately, each consisted of a single genetic cluster (best *K* = 1). There was no particular association between these subpopulations and any of the localities sampled (Figure 2A and Additional file 3). However, each one was associated with the period of the collection. Subpopulation A grouped 173 individuals, 171 (99%) of which were collected in the late rainfall period. Subpopulation B grouped 134 individuals, 110 (82%) of which were collected in the early rainfall period and 24 (18%) in the late rainfall (Figure 2B). The seasonal pattern of alternation between subpopulations A and B from late to early rainfall periods was consistent in all riverine localities, with a predominance of a single subpopulation in both months of collection at each period. In the inland Jaci Paraná, however, subpopulation A predominated over B only in the first month of collection (February), whereas in April, B was already the most abundant (Figure 2A).

The pattern of genetic structuring between sampling periods (i e, early and late rainfall) was confirmed by a significant estimate of global *F*_*ST*_ over loci (0.023, P < 0.0001). When all individuals were included significant *F*_*ST*_ was obtained in two (P2 and P9) out of the eight loci analysed (Table 3). The overall *F*_*ST*_ estimate more than doubled when admixed individuals (i e, those with *qi* < 0.80 for both subpopulations, from STRUCTURE) were removed from the analysis (0.062, P <0.0001), and six out of eight loci showed a significant *F*_*ST*_ (Table 3). Pairwise *F*_*ST*_ estimates among sampling sites within subpopulations (without admixed individuals) ranged from 0 to 0.036 in subpopulation A (with three out of 21 significant comparisons, see Additional file 2), while in subpopulation B *F*_*ST*_ values varied between 0.002 and 0.032 and were all non-significant (Additional file 2).

**Table 3:** **Estimates of***F*_*ST*_**per locus in *Anopheles darlingi *** **populations with and without admixed individuals**

**Locus**	**All individuals**	**No admixed individuals**
ADC28	0,007	0,031
ADC01	-0,001	0,004
ADC110	0,013	0,048
ADC138	0,011	0,054
ADC02	0,003	0,008
ADC137	0,013	0,023
P2	0,032	0,099
P9	0,113	0,241
Over all loci	0,023	0,062

Admixed individuals were determined from STRUCTURE analysis (0.2 < *qi* <0.8). In bold: significant *F*_*ST*_ value after Bonferroni correction.

Furthermore, the component of variance between sampling periods was higher than that among sampling sites within each subpopulation when admixed individuals were excluded, showing 2.87% of genetic variation between sampling periods, 14 times higher than the non-significant percentage of variation among populations within subpopulation (Additional file 4).

The analysis with NewHybrids showed a homogeneity of subpopulation A, associated with the late rainfall sampling period, with 96% of the individuals being assigned as purebred A and 4% as hybrids. In subpopulation B, associated with the early rainfall period, 46% individuals were classified as purebred B, 6% as purebred A and 48% were assigned to hybrid classes (Table 4). Of the four hybrid classes, only F2 hybrids had individuals with *qi* > 0.5. In subpopulation B 39% of individuals were classified as F2, while in subpopulation A 4% of individuals were assigned as F2 hybrids (Table 4).

**Table 4:** **Frequencies of purebred and hybrid individuals, as detected by NewHybrids**[41]**in each of the lineages determined by STRUCTURE analysis**

		**NewHybrids**						
		**Pure-bred A**	**Hybrids**					**Pure-bred B**
STRUCTURE	Lineage A (*N* = 173)	173 (100.0)	0(0.0)					0 (0.0)
	Admixed (*N* = 133)	80 (60.2)	53 (39.8)					0 (0.0)
			F1	F2	Bx. A	Bx. B	Other	
				33 (24.8)			20 (15.0)	
	Lineage B (*N* = 134)	0 (0.0)	48 (35.8)					86 (64.2)
			F1	F2	Bx. A	Bx. B	Other	
				25 (18.7)			23 (17.2)	

*N*: Total number of individuals assigned to each lineage based on the results of STRUCTURE (*qi* > 0.80 for lineage A or B, 0.20 < *qi* < 0.80 for admixed). For each lineage and admixed individuals, the number and percentage (in parenthesis) of individuals assigned to each of the NewHybrids classes (i e, purebred A or B; and F1, F2, and backcrosses [Bx.] with A or B hybrids) with a probability >0.5 is given.

Other: individuals that had a probability of assignment <0.5 for all six classes.

### Subpopulation diversity and demographic inference

Table 5 presents estimates of genetic diversity for subpopulations A and B (excluding admixed individuals). There were no significant differences between subpopulations in the mean allele richness (Wilcoxon signed-ranks test: *P =* 0.575), mean private allele richness (Wilcoxon signed-ranks test: P = 0.575) or expected heterozygosity (Wilcoxon signed-ranks test: *P =* 0.735).

**Table 5:** **Estimates of genetic diversity for lineages A and B (as determined by STRUCTURE) of *Anopheles darlingi***

	**Lineage A**	**Lineage B**
Allele richness (*Rs*)	13.7 (±8.6)	13.4 (±8.5)
Private alleles richness (*Ps*)	3.4 (±2.7)	3.1 (±2.5)
Expected heterozygosity (*He*)	0.766 (±0.198)	0.731 (±0.290)

Values correspond to mean over loci estimates.

In parenthesis: standard deviation of mean.

Since only two individuals were assigned to subpopulation A in early rainfall sampling period, heterozygosity tests implemented in BOTTLENECK were performed on three samples: subpopulation A (late rainfall), subpopulation B (early rainfall) and subpopulation B (late rainfall). Wilcoxon tests revealed a significant deficit of heterozygotes indicative of recent population expansion in subpopulation A while all other tests were non-significant (Additional file 5).

### Mosquito infection rates

Of the total mosquito DNA samples analysed (N = 440), 16 (3.6%) were positive for *Plasmodium sp.* DNA. Infected mosquitoes were found in nine out of the 12 collections analysed. The collections with no positive mosquitoes were Bate Estaca (early rainfall), Vila Candelária (early rainfall) and Teotônio (late rainfall). Infection rates were comparable between the two sampling periods: 3.2% (five out of 158) in the early and 3.9% (11 out of 282) in the late rainfall (χ^2^ = 0.157; d.f. 1; *P* = 0.692). *Plasmodium falciparum* was the predominant species, occurring in 14 (87.5%) of the positive mosquitoes. There was also one mosquito (subpopulation A, late rainfall) infected with *P. vivax* and one *P. falciparum*/*P. vivax* mixed infection (subpopulation A, late rainfall).

Concerning each *A. darlingi* genetic subpopulation, overall *Plasmodium* infection rate was higher in subpopulation A (4.6%) when compared to subpopulation B (3.0%) or admixed individuals (3.0%) but this difference was non-significant (Fisher’s exact test: *P* = 0.692). For subpopulation B and admixed individuals, it was possible to compare infection rates between seasons but no significant differences were found (Table 6). In subpopulation B, there was an increase of infection rates from the early (2.7%) to the late rainfall (4.2%) period (Fisher’s exact test, P = 0.550), while admixed individuals showed an opposite trend (early rain: 4.3%, late rain: 2.3%; Fisher’s exact test: *P* = 0.430).

**Table 6:** **Number of *Anopheles darlingi *** **infected with *Plasmodium sp. *** **per lineage sampling period**

		**Lineage A**	**Admixed**	**Lineage B**
Early rain	*N*	2	46	110
	Pos.	0 (0.0)	2 (4.3)	3 (2.7)
Late rain	*N*	171	87	24
	Pos.	8 (4.7)	2 (2.9)	1 (4.2)
Total	*N*	173	133	134
	Pos.	8 (4.6)	4 (3.0)	4 (3.0)

*N*: sample size. Pos.: number and percentage (in parenthesis) of mosquitoes positive for *Plasmodium sp.* DNA by real-time PCR (see Methods). Lineages A, B and admixed individuals were determined by STRUCTURE analysis.

## Discussion

In Amazonian, suburban, riverine populations of *A. darlingi* near Porto Velho (RO), two genetically differentiated subpopulations associated with different seasonal sampling periods were detected. Subpopulation A is almost exclusively found during or at the end of the rainy season. However, subpopulation A was less abundant in the inland locality (Jaci Paraná) where it appeared to be replaced by subpopulation B earlier in the rainy season than in riverine localities. In this sense, subpopulation A seems to be ecologically closer to a typical forest/riverine *A. darlingi* population that displays the highest mosquito density after the peak of rainfall [18]. In contrast, subpopulation B was the most frequent at the beginning of the rainy season, before *A. darlingi* reaches its late rainy season peak*.* A previous mtDNA-based study conducted in the same region also reported a seasonal difference in the mosquito population of Portuchuelo, a riverine village *ca.* 30 km northeast of Porto Velho, but no seasonal mtDNA differences were found in the other localities analysed [9]. Other studies, using most of the same microsatellite markers here analysed, compared samples from broader geographical ranges (continental scale) and revealed patterns of population structure correlated with geographic distance [15-17]. In these studies, the *F*_*ST*_ estimates among samples at smaller distances (up to 80 km) were comparable with the *F*_*ST*_ estimates within subpopulations obtained in this study. However, none of these microsatellite-based studies compared samples from the same locality at different seasons.

Two hypotheses can be proposed to explain the observed pattern of seasonal genetic variation. The genetic differences between sampling periods may reflect drastic fluctuations in the effective population size of a single genetic entity caused by the transition between rainy and dry seasons. This would agree with the marked seasonal variation in mosquito abundance reported for this region, where during the dry season *A. darlingi* densities decline to residual values [21]. If this was the case then one might expect differences in the level of genetic diversity between subpopulations. Subpopulation B, which prevails at the beginning of the dry season, would display lower levels of genetic diversity and a signal of a recent bottleneck. This was not the case in the present study. Both subpopulations presented similar levels of genetic diversity and a signal of population perturbation (expansion) was detected only in subpopulation A.

The other hypothesis assumes the occurrence of two genetically distinct and partially isolated subpopulations of *A. darlingi*. Subpopulation A would represent a subpopulation more dependent of the rainfall regime, probably adapted to exploit temporary larval habitats in forested, riverine, flooded areas. The bio-ecology of this subpopulation would thus be closer to the typical *A. darlingi*. This would agree with its predominance at the end of the rainy season, where a signal of population expansion was detected and the decline over the dry season as evidenced by its near absence from collections at the early rainfall period. On the other hand, subpopulation B would correspond to a subpopulation more adapted to the dry season, in agreement with its predominance in collections at the onset of the rains. This subpopulation could have derived from the typical subpopulation A through adaptation to more permanent anthropogenic larval habitats, in urban and semi-urban settings. This would result in the maintenance of a resident *A. darlingi* population in urban areas during the dry season with a relatively stable effective population size. In the urban and suburban localities sampled, this population should thus be the first to peak at the start of the rainy season while subpopulation A would peak later in the rainy season when the availability of temporal larval habitats is highest in the forest/riverine flooded areas surrounding these localities.

In the Amazonian region, the variety of larval habitats explored by *A. darlingi* is large, ranging from typical natural breeding sites such as the margins of slow flowing streams, river arms and lagoons, to more human-dependent sites such as irrigation canals, flooded crop fields and mine excavation pools. Such diversity in larval ecology may set a ground for genetic divergence driven by ecological adaptation at the larval stage. This was the case of the recent radiation of the malaria vector *Anopheles coluzzii* (formerly denoted as molecular form M [45]) within the Afrotropical *Anopheles gambiae* complex. This species is considered to be more adapted to permanent breeding sites due to superior predator avoidance [46]. In addition, there is evidence for a recent adaptation of central African populations of *A. coluzzii* to densely urbanized areas by occupation of permanent breeding sites of an anthropogenic nature and a high degree of pollution [47-49]. These studies demonstrate that ecological heterogeneities derived from human-made environmental changes may promote rapid ecological niche adaptation associated with evolutionary divergence in mosquito vectors.

Further studies involving additional mosquito surveys in the region of Porto Velho are required to validate the proposed hypotheses. These studies will also allow verifying if the observed pattern of seasonal genetic partitioning within *A. darlingi* is a stable condition or if it was a sporadic/transient observation. The complete genome sequence of *A. darlingi* has been published recently [5]. This remarkable achievement will result in a much greater availability of polymorphic markers (e g, single nucleotide polymorphism) to study genetic variation of this vector. Combined with appropriate field surveys, it will be possible to obtain a much better clarification of the extent of genetic divergence between subpopulations and its association with local adaptation of *A. darlingi* in the region.

The vast majority of individuals within subpopulation A were classified as pure-bred while the genetic background of subpopulation B was more admixed, with 48% of individuals classified as hybrids. The different proportions of admixture within subpopulations suggest a pattern of asymmetric introgression, with more subpopulation A alleles being introgressed into subpopulation B. This pattern is consistent with the hypothesis of the presence of a resident subpopulation (subpopulation B) in the urban setting that is seasonally exposed to a genetically distinct subpopulation (subpopulation A) promoting local hybridization during the rainy season. It remains to be determined which factors may determine the unbalanced genetic exchange between subpopulations A and B.

Asymmetric introgression is not an uncommon event in mosquitoes. Similar findings have been reported between closely related taxa in *Culex pipiens*[50,51] and *A. gambiae*[52]. These patterns have been related with ecological and behavioural differences between the interbreeding taxa (e g, mating strategies) [49]. While the detection of only F2 hybrids within each subpopulation may suggest that selection is acting against F1 hybrids [53], this result should be interpreted with caution. The Bayesian approach implemented in NewHybrids requires a large number of highly diagnostic markers between populations to accurately assign individuals to hybrid classes [40,53].

From the locus specific *F*_*ST*_ estimates, there were two loci (P2 and P9) that displayed the highest differentiation between subpopulations. These loci may be located near genomic regions under divergent selection that may harbour genes associated with phenotypic differences between the subpopulations that may promote sympatric divergence with gene flow [54,55]. It is noteworthy, however, that these two loci differ from the remaining ones in two aspects: they were isolated from a salivary gland cDNA library [26] and they also differ in the repeat motif (trinucleotides), factors that can affect comparisons with the remainder loci.

The findings of this study suggest that *A. darlingi* appears to be adapting to human modified habitats by deviating its breeding preferences into more permanent larval habitats, which may lead to an increased persistence during the dry season. This adaptation may have important consequences in regional malaria transmission patterns. Both subpopulations were found infected with malaria parasites at comparable rates, with the highest values occurring at the end of the rainy season, in accordance with the seasonal patterns of malaria transmission intensity characteristic of the Amazonian region [56]. However, subpopulation B was also found to be infected by malaria parasites in the early rainfall period, and with considerable infection rates. Moreover, admixed individuals had a higher infection rate in the period after the dry season. Although the presence of parasite DNA in a mosquito does not fully incriminate it as infective (only presence of sporozoites in salivary glands), it is still an indicator of the potential for malaria transmission given the recognized vector competence of *A. darlingi*. The co-occurrence of both subpopulations in this region may thus promote a more perennial transmission of malaria throughout the year, with obvious implications for malaria epidemiology and control.

Several studies have demonstrated that deforestation and human colonization of deforested areas have a strong impact in mosquito populations, with an increase in larval breeding and human-biting activity [7,57,58] thereby increasing malaria risk [59,60]. Moreover, the increased landscape disturbance resulting from the construction of two hydroelectric power plants in the Madeira River may promote greater changes in the genetic structure and bio-ecology of this malaria vector, with undetermined consequences for malaria transmission [61]. In this context, the results of this study may serve as a baseline for future bio-ecological and genetic monitoring of *A. darlingi* populations in this malaria endemic region.

## Conclusions

The co-occurrence of two genetically distinct subpopulations of *A. darlingi* adapted to different periods of rainfall may promote a more perennial transmission of malaria throughout the year. These findings in a context of strong environmental impact due to deforestation and dam construction preclude serious implications for malaria epidemiology and control in the Amazonian region.

## Abbreviations

mtDNA: Mitochondrial DNA; INMET: Instituto Nacional de Meteorologia; PCR: Polimerase Chain Reaction; rRNA: Ribosomal ribonucleic acid; dNTPs: Deoxynucleoside triphosphate; SMM: Stepwise Mutation Model; TPM: Two Phase Model; He: heterozygosity, heterozygosity (He) allele richness (Rs) and private allele richness (Ps).

## Competing interests

The authors declare that they have no competing interests.

## Authors’ contributions

All the authors collaborated on the work presented in this study. AFA and PEMR defined the research theme; LHSG was responsible for the mosquitoes collection; AFA designed the methods, performed the experiments and interpreted the results; JLV performed some of the experiments; AFA, PS, JP, and PEMR analysed the data and drafted the manuscript. All authors read and approved the final version of the manuscript.

## Supplementary Material

Additional file 1 **Title: Repeats and primer sequences of the microsatellite loci designed for *A. darlingi.*** Description: Microssatelites used to genotype *Anopheles darlingi*, Locus position, numer of repeats, primer sequence , Tm of the reaction and fluorechrome of each *loci* used. Click here for file

Additional file 2 **Title: Estimates of pairwise genetic differentiation among localities in *A. darlingi *** **pure subpopulation (without admixed): A) subpopulation A, B) subpopulation B.** Description: Pairwise genetic differentiation of grouped subpopulations A and B excluding admixture individuals. Click here for file

Additional file 3 **Title: Relative distribution of each genetic lineage revealed by STRUCTURE per locality/semester.** Description: A table showing the distribution of subpopulation A, subpopulation B and admixture population at different collection sites and period. Click here for file

Additional file 4 **Title: Apportionment of molecular variance measured among populations of *Anopheles darlingi *** **from all samples, or between populations from the two semesters.** Description: Molecular variance of samples considering subpopulation A and subpopulation B. Click here for file

Additional file 5 **Title: Summary of BOTTLENECK tests.** Description: List of Bottleneck tests at Subpopulations A and B at each period of collection. Click here for file
