# Supplementary material for: Seasonal genetic partitioning in the neotropical malaria vector, Anopheles darlingi
Source: Malar J. 2014 May 29;13:203. doi: 10.1186/1475-2875-13-203 (PMC4059831; doi:10.1186/1475-2875-13-203)
Supplement: Additional file 1 — Title: Repeats and primer sequences of the microsatellite loci designed for A. darlingi. Description: Microssatelites used to genotype Anopheles darlingi, Locus position, numer of repeats, primer sequence , Tm of the reaction and fluorechrome of each loci used. [file 1475-2875-13-203-S1.docx]

## Table S1.

**Table S1. Repeats and primer sequences of the microsatellite loci designed for *A. darlingi***

| **Locus**  **(GenBank)** | **Repeat**  **numbers** | **Primer sequences** | **Ta(°C)** | **Fluorochrome** |
| --- | --- | --- | --- | --- |
| ADC01  (AF322185) | (GA)_21_ | f-CGACACCGCACTTGCTACTACCTG  r-GCTCTAATGATGCTCGTAACCGCT | 55 | FAM |
| ADC02  (AF322186) | (GA)_7+7_ | f-TACCACTGCGTCCTTAGACACTG  r-CACACTGGGGCATCATTCATTTC | 52 | FAM |
| ADC28  (AF322187) | (GA)_9_ | f-TGCCCATCCACTGCGTAACGG  r-CTCGTCGTCAGCGTCGTGC | 56 | FAM |
| ADC29  (AF322188) | (GA)_22_ | f-AATCACGGTGCACGATGC  r-GATCGTTGGCCGAGAATG | 56 | NED |
| ADC110  (AF322190) | (GT)_16_ | f-CGTTCGACACAATCGTTACCACG  r-CCGAACAACAGCCAACAGCTGTG | 54 | HEX |
| ADC137  (AF322191) | (GT)_1_ | f-CAGCCACCCATACGCTGTTGACCA  r-TCTTACGGGAATGGTGCGACGCTC | 56 | HEX |
| ADC138  (AF322192) | (AC)_14+3_ | f-CCATTCTCGCAGCCTCCAGGAC  r-CTTTGAGCCGGTGCTGTGCTGC | 57 | NED |
| ADC107n*  (AF322189) | (AC)_12_ | f-GTCCACTCCCAGGCACAC  r-AGCAATCGAGGCAAACTTTC | 56 | HEX |
| ADSP2*  (DV729944) | (GTA)_7_ | f-GCATATATTCTCGCCGCATT  r-TCAGCTACTACCCGACGACA | 53 | FAM |
| ADMP9*  (DV729762) | (AAC)_11_ | f-ACAACGTCCAATGCAACAAC  r-CTCGAGGGCTTTCTGTATCG | 57 | HEX |

*Unpublished primers. Ta: annealing temperature. The GenBank accession number is listed below the corresponding locus.
