# Supplementary material for: Seasonal genetic partitioning in the neotropical malaria vector, Anopheles darlingi
Source: Malar J. 2014 May 29;13:203. doi: 10.1186/1475-2875-13-203 (PMC4059831; doi:10.1186/1475-2875-13-203)
Supplement: Additional file 2 — Title: Estimates of pairwise genetic differentiation among localities in A. darlingi pure subpopulation (without admixed): A) subpopulation A, B) subpopulation B. Description: Pairwise genetic differentiation of grouped subpopulations A and B excluding admixture individuals. [file 1475-2875-13-203-S2.docx]

## Table S3.

**Table S3 Estimates of pairwise genetic differentiation among localities in *A. darlingi* pure subpopulations (without admixed): A) subpopulation A, B) subpopulation B**

| **Table S3 –A** | **Teotônio 1** | **Santo Antônio 1** | **Jaci Paraná 1** | **Engenho Velho 1** | **Vila Candelária 1** | **Bate Estaca 1** |
| --- | --- | --- | --- | --- | --- | --- |
| **Teotônio 1** | - |  |  |  |  |  |
| **Santo Antônio 1** | 0*.*011 | - |  |  |  |  |
| **Jaci Paraná 1** | -0*.*009 | -0*.*012 | - |  |  |  |
| **Engenho Velho 1** | 0*.*027 | **0*.*035** | 0*.*020 | - |  |  |
| **Vila Candelária 1** | -0*.*011 | **0*.*027** | 0*.*007 | 0*.*028 | - |  |
| **Bate Estaca 1** | 0*.*012 | 0*.*007 | -0*.*010 | **0*.*036** | 0*.*010 | - |
| **Amazonas 1** | -0*.*004 | 0*.*008 | -0*.*015 | 0*.*027 | -0*.*004 | -0*.*010 |

**Bold** indicates a significant value after Bonferroni correction, **1-** sampling sites from the first semester (late rainy season)

| **Table S3 –B** | **Santo Antônio 2** | **Jaci**  **Paraná 2** | **Engenho**  **Velho 2** | **Vila**  **Candelária 2** | **Bate**  **Estaca 2** |
| --- | --- | --- | --- | --- | --- |
| **Santo Antônio 2** | - |  |  |  |  |
| **Jaci Paraná 2** | 0*.*018 | - |  |  |  |
| **Engenho Velho 2** | 0*.*008 | 0*.*016 | - |  |  |
| **Vila Candelária 2** | 0*.*018 | 0*.*031 | 0*.*006 | - |  |
| **Bate Estaca 2** | 0*.*002 | 0*.*026 | 0*.*017 | 0*.*002 | - |
| **Jaci Paraná 1** | 0*.*016 | 0*.*032 | 0*.*007 | 0*.*001 | 0*.*008 |
| **1-** sampling sites from the first semester(late rainy season), **2-** sampling sites from the second semester(early rainy season) | | | | | |
