# Supplementary material for: Seasonal genetic partitioning in the neotropical malaria vector, Anopheles darlingi
Source: Malar J. 2014 May 29;13:203. doi: 10.1186/1475-2875-13-203 (PMC4059831; doi:10.1186/1475-2875-13-203)
Supplement: Additional file 3 — Title: Relative distribution of each genetic lineage revealed by STRUCTURE per locality/semester. Description: A table showing the distribution of subpopulation A, subpopulation B and admixture population at different collection sites and period. [file 1475-2875-13-203-S3.docx]

## Table S2.

| **Table S2 Relative distribution of each genetic lineage revealed by STRUCTURE per locality/semester** | | | | | |
| --- | --- | --- | --- | --- | --- |
|  |  | **Subpopulation A** | **Subpopulation B** | **Admixed** | **Total** |
| **Total** | **N** | 173  39% | 134  31% | 133  30% | 440 |
| **1st semester** | **Total Semester 1** | 171 | 24 | 87 | 282 |
|  |  | 61% | 9% | 31% |  |
|  | **Vila Candelária 1** | 30 | 1 | 12 |  |
|  |  | 70% | 2% | 28% |  |
|  | **Bate Estaca 1** | 28 | 2 | 18 |  |
|  |  | 58% | 4% | 38% |  |
|  | **Santo Antônio 1** | 34 | 1 | 13 |  |
|  |  | 71% | 2% | 27% |  |
|  | **Engenho Velho 1** | 23 | 1 | 19 |  |
|  |  | 53% | 2% | 44% |  |
|  | **Teotônio 1** | 22 | 0 | 9 |  |
|  |  | 71% | - | 29% |  |
|  | **Amazonas 1** | 16 | 1 | 4 |  |
|  |  | 76% | 5% | 19% |  |
|  | **JaciParaná 1** | 18 | 18 | 12 |  |
|  |  | 38% | 38% | 25% |  |
| **2nd semester** | **Total Semester 2** | 2 | 110 | 46 | 158 |
|  |  | 1% | 70% | 29% |  |
|  | **Vila Candelária 2** | 0 | 22 | 13 |  |
|  |  | - | 63% | 37% |  |
|  | **Bate Estaca 2** | 0 | 18 | 13 |  |
|  |  | - | 58% | 42% |  |
|  | **Santo Antônio 2** | 1 | 22 | 6 |  |
|  |  | 3% | 76% | 21% |  |
|  | **Engenho Velho 2** | 1 | 33 | 5 |  |
|  |  | 3% | 85% | 13% |  |
|  | **JaciParaná 2** | 0 | 15 | 9 |  |
|  |  | - | 63% | 38% |  |
| N, number of individuals; Values in percentage refer to the frequencies within each lineage. Individual genetic assignment to lineages was based on a minimum posterior probability threshold (Tq) of 0.80. Individuals displaying 0.20<q <0.80 were considered of admixed ancestry | | | | | |
