# Supplementary material for: Seasonal genetic partitioning in the neotropical malaria vector, Anopheles darlingi
Source: Malar J. 2014 May 29;13:203. doi: 10.1186/1475-2875-13-203 (PMC4059831; doi:10.1186/1475-2875-13-203)
Supplement: Additional file 4 — Title: Apportionment of molecular variance measured among populations of Anopheles darlingi from all samples, or between populations from the two semesters. Description: Molecular variance of samples considering subpopulation A and subpopulation B. [file 1475-2875-13-203-S4.docx]

## Table S4.

| **Table S4 Apportionment of molecular variance measured among populations of *Anopheles darlingi* from all samples, or between populations from the two semesters** | | | | | | | |
| --- | --- | --- | --- | --- | --- | --- | --- |
|  | **1 group (all samples)** | |  | **2 groups (2 seasons)** | | | |
|  | Among populations within group | |  | Between groups | | Among populations within group | |
|  | % variation | P |  | % variation | P | % variation | P |
| **All individuals** | 0.99 | <0.0001 |  | 0.91 | <0.001 | 0.52 | <0.01 |
| **No admixed individuals** | 2.27 | <0.0001 |  | 2.87 | <0.0001 | 0.23 | N.S. |
| **Subpopulation A**  **(without admixed)** | 0.45 | N.S. |  | - | - | - | - |
| **Subpopulation B**  **(without admixed)** | 0.95 | N.S. |  | - | - | - | - |
| Admixed individuals were determined from the previous STRUCTURE analysis (0.2<q <0.8). P represents the significance of the variation | | | | | | | |
