# Supplementary material for: Seasonal genetic partitioning in the neotropical malaria vector, Anopheles darlingi
Source: Malar J. 2014 May 29;13:203. doi: 10.1186/1475-2875-13-203 (PMC4059831; doi:10.1186/1475-2875-13-203)
Supplement: Additional file 5 — Title: Summary of BOTTLENECK tests. Description: List of Bottleneck tests at Subpopulations A and B at each period of collection. [file 1475-2875-13-203-S5.docx]

## Table S5.

| **Table S5**  **Summary of BOTTLENECK tests** | | | |
| --- | --- | --- | --- |
|  |  | **SMM** | **TPM (95%)** |
| **Subpopulation A-late rain** | *P*_(_*_He_* _>_ *_Heq_*_)_ | N.S. | N.S. |
|  | *P*_(_*_He_* _<_ *_Heq_*_)_ | 0.002 | 0.004 |
| **Subpopulation B-early rain** | *P*_(_*_He_* _>_ *_Heq_*_)_ | N.S. | N.S. |
|  | *P*_(_*_He_* _<_ *_Heq_*_)_ | N.S. | N.S. |
| **Subpopulation B-late rain** | *P*_(_*_He_* _>_ *_Heq_*_)_ | N.S. | N.S. |
|  | *P*_(_*_He_* _<_ *_Heq_*_)_ | N.S. | N.S. |
| Probabilities of heterozygosity deficit or excess (P-value) for each population according to TPM and SMM. He < Heq: Wilcoxon test for heterozygosity deficit, He > Heq: Wilcoxon test for heterozygosity excess. | | | |
